# The Transition of Care From Pediatric to Adult Health-Care Services of Vertically HIV-Infected Adolescents: A Pilot Study

**DOI:** 10.3389/fped.2020.00322

**Published:** 2020-07-02

**Authors:** Grazia Isabella Continisio, Andrea Lo Vecchio, Francesca Wanda Basile, Carla Russo, Maria Rosaria Cotugno, Giulia Palmiero, Cinzia Storace, Carmela Mango, Alfredo Guarino, Eugenia Bruzzese

**Affiliations:** ^1^Section of Pediatrics, Department of Translational Medical Sciences, University of Naples Federico II, Naples, Italy; ^2^Section of Infectious Diseases, Department of Clinical Medicine and Surgery, University of Naples Federico II, Naples, Italy

**Keywords:** HIV, adolescents, transition, biopsychosocial approach, well-being

## Abstract

**Objective:** Clinical and psychological HIV-related problems peak during adolescence, which coincides with transition of children and adolescents infected from mothers from pediatric to adult reference centers for HIV infection. Transition often is done without specific programs. We wanted to explore transition as an opportunity to increase the efficacy of care and the psychological well-being through a specific program.

**Methods:** Thirteen vertically infected patients aged 13–20 years were followed up for 24 months by pediatricians, infectious disease specialists, a psychologist, and a nurse. Interventions consisted in joint clinic, simplification of therapy, patient group discussions, HIV infection explanations, and psychological support, lasting 12 months. Efficacy was measured by viro-immunological outcomes and adherence to therapy and psychological tests. Clinical, viro-immunological, and psychological evaluations were performed at 0 (T0) and 12 months (T12) and 6 months after transition to an adult center (T18). Psychological outcomes were assessed using standardized questionnaires for quality of life and self-esteem.

**Results:** In 11/13 participants, pills administrations/day were significantly reduced. Patients with undetectable viral load and CD4+ >25% increased from 61 to 77% and from 61 to 74%, respectively. Six months after transition, all patients exhibited an undetectable viral load. Adolescents' awareness of the severity of the disease and the risk of sexual transmission was generally poor. Patients classified with “severe” psychological distress according to the quality of life index decreased from 38 to 15% and well-being increased. Similar results were observed 6 months after the transition to adult care. No effect was observed on self-esteem index.

**Conclusions:** Specific protocols for transition should be developed to optimize resilience and psychological well-being, including routine psychological support for adolescents with HIV infection transiting from pediatric to adult centers for HIV infection.

## Introduction

An increasing number of vertically HIV-infected adolescents are referred to centers for adult (1). In pediatric HIV infection, as in many other childhood illnesses, the chronicization of the disease has led to new problems including adherence, life-long treatment self-management of the medication side effects, stigma, and—of major importance—awareness of sexual transmission (2, 3). The number of drugs approved for pediatric patients is limited compared with that for adults, and this may hamper adherence with antiretroviral therapy (ART), because non-adherence is often associated with side effects and psychosocial implications in children and adolescents (4–6). Moreover, psychological and social HIV-related problems are amplified in children and adolescents growing to adulthood (7–9). In a previous study, using the International Classification of Functioning, Disability and Health instrument (ICF), we found that psychosocial issues had a major impact on quality of life than had clinical problems in a population of children and adolescents with HIV infection (10). In addition, the stigma associated with HIV infection is a major barrier to social and psychological health (11). Sexual transmission of HIV infection inhibits the development of an emotional life in adolescents, affecting the psychosocial stability and the sexual health (12).

All these factors complicate the management of teenagers with HIV infection and negatively impact adherence to ART, medical visits, and psychosocial functioning. Up to 20% of pediatric patients with HIV infection show poor short-term adherence (within the last month) to ART, and this percentage increases to 50% when adherence is assessed in the long term (within the last 3 months) (13–15). The complexity of therapy is one of the major determinants of low adherence in children with HIV. Current guidelines highlight the importance of tailored strategies to implement ART adherence, particularly in adolescents (https://aidsinfo.nih.gov/guidelines/html/1/adult-and-adolescent-arv-guidelines/30/adherence-to-art). Reduction of pill number and administrations per day to once per day fixed dose combination scheme is approved for adult patients only.

The transition to centers for HIV care of adults represents a bridge between the dependence and protection typical of pediatric patient and the liberty, responsibility, and self-management of adult patients and is characterized by specific problems and emotional–relational behaviors in a dynamic equilibrium. However, transition to adult health-care settings is only one of the aspects of the more general growth to adulthood, during which younger people undergo a change that is both systemic and cultural. Although specific programs for transition to adult care centers have been reported for cystic fibrosis, diabetes mellitus, and other chronic diseases, only limited data are available on the transition of adolescents with HIV (16). Transition is associated with several problems and risks, including loss to follow-up, low adherence, disruption of psychological health, problems in hiding HIV status, stigma, and isolation from peers. Therefore, transition is a delicate period in which management changes, and this begs risks for adolescents. Yet transition is often achieved without a specific program, and adolescents are simply referred to centers for adult patients, where management and approach are different from those of pediatric centers. We wanted to explore transition as an opportunity to increase the efficacy of care and the psychological well-being to ultimately increase resilience, a major resource for HIV-infected patients.

The American Academy of Pediatrics states that health-care providers should develop a formal process for transitioning HIV-infected adolescents to adult health care between the ages of 18 and 25, starting with the patients in their early teens (17). However, in most centers for pediatric HIV infection, the patients are either transferred to adult care without a specific program or continue their follow-up in pediatric infectious disease units.

The Transition of Seropositive Children to Adulthood (TOSCA) project was a pilot study carried out to test the efficacy of an integrated multifaceted intervention aimed at optimizing the transition of care from pediatric to adult health-care services. We hypothesized that a specific medical and psychological intervention applied during transition of care to the adult care center improves the viro-immunological and psychological conditions of adolescents, ultimately increasing their resilience.

## Methods

### Study Protocol and Population

The Reference Center for Pediatric HIV/AIDS of the University of Naples Federico II covers a territory of about 7 million inhabitants of Southern Italy and manages about 40–50 HIV-infected children and adolescents with one to three new cases of vertical HIV infection every year.

All patients aged >13 and <20 years who already had received the disclosure of HIV infection and for whom informed consent had been obtained were included in the present study. The study protocol included three phases: in the first phase, we created the multidisciplinary team and developed the study materials. All patients underwent full clinical, immunological, and virological evaluations. In addition, information regarding disease knowledge, adherence to therapy, and psychological status (including self-esteem) was obtained. Next, all patients underwent the bundle of clinical and psychological interventions as detailed below. All the parameters were recorded at baseline (T0) and 12 (T12) months after the preparation to transition was achieved. In the third phase, patients were transferred to adult care. All the parameters were recorded again 6 months after the transition (T18).

The intervention consisted in a bundle of initiatives run by a multidisciplinary group including a pediatrician specialist (PID) as well as an infectious disease specialist (AID), a dedicated nurse, a psychologist, and a social assistant. The group met periodically to discuss study protocol, progresses, barriers, and interventions.

All patients enrolled had been followed up since birth or the initial diagnosis of vertical HIV infection. Before this study, PID took care of HIV-infected adolescents up to 20 years of age in specific pediatric reference and then referred them to AID in the reference center for adult HIV-infected patients. Transition only included a brief discussion and written reports of their clinical and immunological state, previous ART regimen, concomitant diseases, and adherence to ART by the PID specialist.

## Outcome Parameters and Intervention Bundle

### Outcome Parameters

According to the study protocol, the clinical and psychological evaluations were performed before starting the intervention (T0), after 12 months (T12) months, which is during transition, and 6 months after transition.

#### Viro-Immunological Parameters

Every 2 months, each patient with HIV underwent full evaluation of viral load (HIV RNA measured as the number of HIV copies/ml of blood) and of CD4^+^ cell count (measured as the number of CD4^+^ cells/mm^3^ of blood). Both parameters are markers of infection status and of ART efficacy. The same parameters were recorded at T0, T6, T12, and T18 to have a closer control of the state of infection.

#### Patient's Knowledge of HIV Infection

To assess the knowledge of HIV infection, a brief questionnaire was developed with six multiple-choice questions, investigating awareness of modality of HIV transmission and importance of ART. The questionnaire was administered at the beginning of the study and at T12. Each correct response was scored 1, and a wrong response was scored 0.

After baseline assessment, each patient was asked to participate to a discussion on HIV infection, transmission, and management. This was repeated during the intervention period at least two times, and at the end of the intervention, the questionnaire was administered again.

#### Evaluation of Psychological Well-Being

Two standardized questionnaires were used to investigate patients' well-being and self-esteem:

- The *Psychological General Well-Being* (*PGWB*) index is an established patient-reported instrument, which provides self-reported assessment of psychological health. It does not include an evaluation of physical health. This 22-item instrument includes six domains: Anxiety, Depressed Mood, Positive Well-being, Self-Control, General Health, and Vitality. The 22 items generate an overall index or total score for general well-being. The original score for each item ranges from 0 to 5, giving a possible total score 0–110 (18). A total score <59 indicates severe distress, 60–72 moderate distress, and 73–98 no distress. Higher scores indicate higher levels of well-being as perceived by the patient. PGWB takes 10 min or less to complete and is generally well-accepted.
- The *Multidimensional Self-Esteem Test* (*TMA*) is a validated questionnaire for self-esteem particularly in adolescents in its multiple dimensions. The test includes six areas (personal, school, emotional, skills, family, and body), and consists of six groups of 25 items for each area with each item requiring one of four possible answers: absolutely true, true, not true, and absolutely not true. The average scores for self-esteem in a standard reference sample range between 85 and 115 (19).

## Intervention Bundle

### Joint Pediatric/Adult Clinic

A joint transition outpatient clinic was set up by PID and AID physicians and a study nurse in order to have a joint medical visit every 2 months. The last two medical visits for each patient were held at the Department of Adult Infectious Diseases, with the presence of PID, in order to achieve a smooth transition. In the last visit, PID and AID jointly discussed each patient's treatments and simplification of ART regimen in the presence of the patient and his/her family.

### Knowing HIV Infection, Its Transmission Routes, Its Consequences, and Self-Management

A 30- to 60-min face-to-face education session focusing on HIV infection, its clinical manifestations, risks, transmission routes and prevention measures, and principles of ART and adherence was provided to each patient by a PID, and it took place at the Department of Pediatrics. The meeting included time for questions and answers. At the end of session, the physician scored the availability/involvement of each patient using a 5-point Likert scale (1 = strongly disagree to 5 = strongly agree). In order to better understand patients' doubts and requests, overcome barriers linked to anxiety and embarrassment, and drive future meetings, PID specialists and the nurse also answered questions collected from patients, written anonymously and collected in a box in a blinded fashion.

### Psychological Support and Group Discussion: Resilience as a Resource

A psychologist experienced in the management of pediatric HIV infection organized individual meetings once a month; in addition, group meetings with all patients and their families were organized twice a year. A study nurse and a gynecologist took part in these meetings to provide information on prevention of HIV transmission and protection during sexual contacts.

A bundle of interventions and their time points are reported in Table 1.

**Table 1:** Bundle of interventions and their time points.

**Intervention**	**T0**	**T6**	**T12**	**T18**
**Phase 1**				
Informed consent	X			
Development of multidisciplinary team	X			
Development of study material and database	X			
Patient enrollment	X			
**Phase 2**				
Clinical evaluation	X	X	X	X
Assessment HIV immunological class	X		X^*^	X
Assessment CD4+ count and percentage	X	X	X	X
Assessment HIV viral load	X	X	X	X
Educational session	X	X	X	
ART review and simplification		X	X	
Administration of knowledge questionnaire	X		X	
Psychological interview	X		X	
Administration of PGWB questionnaire	X		X	X
Administration of TMA questionnaire	X		X	
**Phase 3**				
Transition of adolescents to AID unit				X
Psychological follow-up				
Analysis of results				X

*ART, antiretroviral therapy; PGWB, Psychological General Well-Being; TMA, Multidimensional Self-Esteem Test*.

* *Or in case of any acute illness defining severe AIDS*.

## Statistical Analysis

Quantitative variables were reported as means ± standard deviation (*SD*), and variables with skewed distributions were presented as medians and interquartile ranges (IQRs). Results were compared by *t*-test or the Mann–Whitney non-parametric test, as appropriate. Categorical variables were summarized and reported as frequencies and percentages and compared by Fisher's exact test or chi-square test, as appropriate.

Two-sided *p* < 0.05 were considered statistically significant. Statistical analysis was performed using the SPSS software (version 20.0 for Windows; SPSS Inc., Chicago, IL, USA).

## Data Masking, Ethics, and Financial Support

Single codes were assigned to each enrolled patient to collect and report data anonymously. All study data were collected through manual chart and reviewed by two persons (ALV and EB) involved in the study and recorded in a Microsoft Excel database. The results of psychological tests were analyzed by a psychologist and a social worker and subsequently recorded in the same database. At enrollment, the study protocol was presented and discussed with patients and caregivers who signed a specific informed consent according to patients' age.

The study was conducted according to the Declaration of Helsinki, and the protocol was approved by the Ethical Committee of the University Federico II of Naples (protocol number 229/15). The TOSCA study was supported by a competitive grant by GILEAD Sciences S.r.l. (Milan, Italy).

## Results

### Study Population

The eligible population included 13 adolescents with vertical HIV infection on ART followed up to the reference center of Naples. All patients (age range 14–20 years) were enrolled. The general characteristics of the population at enrollment are shown in Table 2.

**Table 2:** General characteristics of the study population and HIV class at study enrollment.

**Characteristics**	***N***
Total enrolled patients (male/female)	13 (5/8)
Median age at diagnosis, years (range)	2 (1–10)
Median age at enrollment, years (range)	17 (14–20)
**Ethnicity**	
Caucasian	11/13
African	2/13
Years on ART, mean (SD)	14 (3)
HIV disease and infection class^*^at diagnosis	***n***
Class (C1/C2/C3)	0/0/1
Class (B1/B2/B3)	1/1/6
Class (A1/A2/A3)	3/1/0
**Patients with undetectable HIV viral load^§^**	8

*ART, antiretroviral therapy*.

* *HIV disease staging is defined according to the U.S. Centers for Disease Control and Prevention (CDC) pediatric classification system*.

§ *Defined as HIV RNA <40 copies/ml*.

During the study, one patient developed a severe psychiatric disorder and dropped out before the end of transition process (T12). For this patient, no data are available for T18.

### Modifications of Antiretroviral Therapy

**Figure 1:**
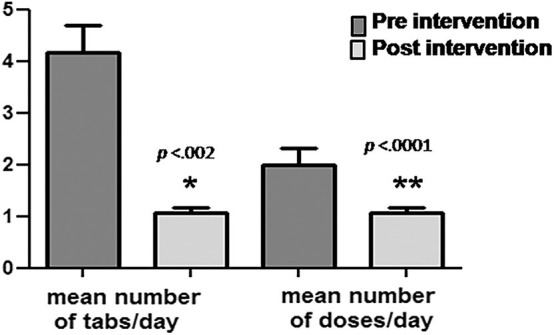
Simplification of therapy by applying the drug protocols available for adult patients. Both the mean number of daily pills and number of daily drug doses were significantly reduced (4.18 ± 1.72 to 2.0 ± 1.1, *p* < 0.002, and 1.82 ± 0.4 to 1.09 ± 0.3, *p* < 0.0001, respectively) during the transition period. *p < 0.005; **p < 0.0001.

In 11/13 participants (85% of the total), the pediatric drug regimen was simplified by adopting the adult protocol during the 12 months of transition protocol. Both the number of daily pills and doses were significantly reduced (from 4.18 ± 1.72 to 2.0 ± 1.1, *p* < 0.002, and from 1.82 ± 0.4 to 1.09 ± 0.3, respectively, *p* < 0.0001). For three of the nine patients (33.3%) on a multi-tablet regimen, it was possible to switch to once-daily fixed dose combination scheme (Figure 1).

### Viro-Immunological Parameters

At 12 months post-intervention, the total number of patients with undetectable viral load in blood increased. An increase in CD4+ lymphocytes was documented at T12, when 11/13 patients showed a percentage of CD4+ above 25%. At 18 months, all transited patients were viro-suppressed, and an increase of mean CD4+ cell count was observed, although the number of patients with a percentage of CD4+ above 25% slightly decreased. The results of HIV viral load and of the CD4+ lymphocytes count at T0, T6, T12, and T18 are reported in Table 3.

**Table 3:** Viral and immunological modifications observed in HIV-infected adolescents at baseline, during, and 6 months after the intervention.

**Characteristics**	**T0 (*n* = 13)**	**T6 (*n* = 13)**	**T12 (*n* = 13)**	**T18 (*n* = 12)**
**HIV viral load**			
HIV RNA <40 copies/ml (*n*, % of patients)	8 (61.5)	8 (61.5)	10 (77)	12 (100)
**Immunological status**			
T CD4+ <15% (*n*, %)	1 (7.7)	0 (0)	0 (0)	0 (0)
T CD4+ 15–25% (*n*, %)	4 (30.8)	4 (30.8)	2 (15.4)	3 (25)
T CD4+ > 25% (*n*, %)	8 (61.5)	9 (69.2)	11 (84.6)	9 (75)
T CD4+ absolute count (mean ± SD)	711 ± 357	728 ± 338	724 ± 297	799 ± 411

### Knowledge of HIV

In the pre-intervention evaluation, none of the patients was able to correctly answer all the questions. However, at the end of the project, 3/13 patients gave all the correct answers to the six questions in the questionnaire (0 vs. 23%). On average, the mean number of correct answers, at baseline, was 3.62 ± 1.39; at the end of the study, this number increased to 4.31 ± 1.38 (*p*.21). Of note, 5/13 (38%) patients answered “I do not know” to the question: “Based on current knowledge, can you recover from HIV infection?” Furthermore, eight patients (61%) reported that they did not consider the therapy useful to protect partners or close contacts from being infected. Surprisingly, all patients showed a high level of willingness to participate in the assessment sessions and the discussion, both in the pre-intervention and post-intervention phases; and the number of missed meetings was limited.

### Psychological Interventions

A total of 25 psychological interviews and 25 PGWB and 23 TMA tests were completed. With regard to the psychological interviews, most of the patients showed an adequate level of participation as judged by the timeliness and the adherence to the scheduled meetings. The patients reported their personal experience of living with a chronic infectious disease, their relationships with peers, and the psychological burden of the disease and its therapy. Individual experiences, personal worries, and possible solutions were shared with the group and discussed. The concept of “transition” was extensively discussed as an active passage to adulthood and a necessary step to self-management of HIV infection, but also as an opportunity to effectively address unresolved issues.

**Figure 2:**
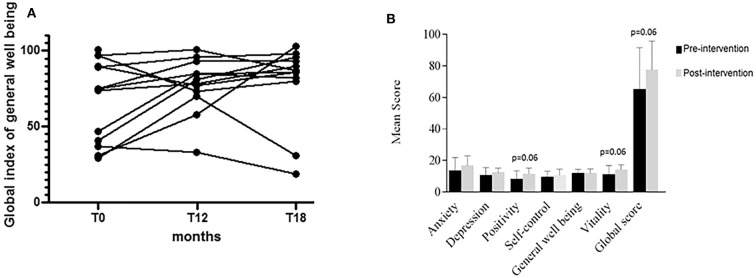
Assessment of general psychological well-being. **(A)** At baseline assessment, a “severe” level of distress was found in 5 of 13 patients (38%), with scores lower than 59. At the end of intervention (T12), nine of the 12 remaining patients showed an increase in the PGWB scores compared with the baseline pattern, but in three cases, the scores were lower compared with baseline. One patient, who showed a condition of severe distress at baseline, improved at the end of the intervention but returned to his/her basal condition as early as 6 months after transition. One patient showed a slight deterioration of the well-being index during the intervention, which further worsened after transition. **(B)** The score increase was more pronounced in the two specific domains of vitality and positivity. The global score of general well-being increased at the end of intervention, although the value did not reach statistical significance (*p* = 0.06).

All patients answered the PGWB and TMA questionnaires, showing good levels of participation and cooperation. As shown in Figure 2A, at baseline assessment, a “severe” level of distress was found in 5 of the 13 patients (38%), with scores lower than 59. Seven other patients with scores between 73 and 98 and 1 > 100 showed no distress or even positive well-being on PGWB. After 12 months, only two patients (15%) were still in the severe distress range, one showed moderate distress, and the remaining nine had no distress. Nine out of twelve patients showed an increase in the global wellness index (PGWB) scores compared with the baseline, but in three cases, the scores were lower compared with baseline. Surprisingly, one patient with severe distress at baseline improved at the end of the intervention but returned to his basal condition already 6 months after transition. One patient showed a slight deterioration on the well-being index during the intervention and showed a further slight worsening after the transition (Figure 2A). The global score of general well-being increased at the end of intervention from 68 to 77.5, although the difference did not reach statistical significance (*p* = 0.06). The increase was more pronounced in the two specific domains of vitality and positivity (Figure 2B). Six months after transition, the mean global score of general well-being was 79, indicating no distress.

**Figure 3:**
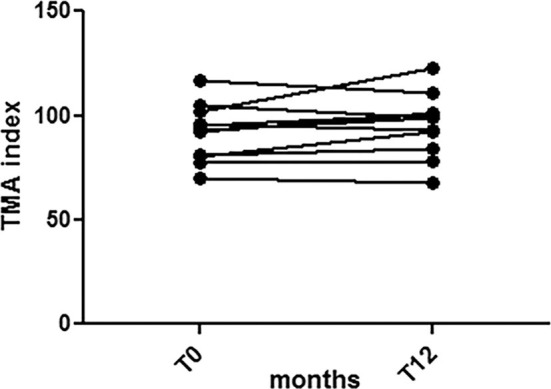
Self-esteem assessment. Seven out eleven patients with HIV infection reached or exceeded a score of 85 at T0. After 12 months of intervention, 8/11 had a score higher than 85. Of the four patients with an initial score below 85, only one patient scored more than 85 at the 12-month follow-up; the other three did not show any improvement in self-esteem. Overall, the results showed that HIV-infected adolescents exhibited a good level of self-esteem.

Eleven of the 13 patients also completed the two-point evaluation of TMA (T0 and T12). This test showed that the majority of patients (7/11) with HIV infection had a fair level of self-esteem, having reached or exceeded a score of 85 at T0. After 12 months of intervention, 8/11 patients had a score higher than 85. Of the four patients with an initial score below 85, only one patient scored more than 85 at the 12-month follow-up; the other three did not show any improvement of self-esteem (Figure 3).

## Discussion

Transition provides an opportunity to improve clinical and psychological outcome and increase their resilience, and we observed that viro-immunological parameters as well as psychological health improved or showed an improving trend upon application of multifaceted clinical and psychological intervention.

In children and adolescents, ART is effective in controlling HIV-related symptoms; but the overall quality of life is far from optimal, owing to problems in the psychosocial sphere (10). In a previous study, we observed a major burden of psychosocial issues in a population of HIV-infected compared to a population of non-HIV-infected children (20). Poor socioeconomic conditions, high rates of unemployment, family breakdown, and problems in HIV disclosure are the most difficult obstacles in the management of HIV-infected adolescents (21). This indicates that psychosocial parameters, rather than just the clinical aspects, should be a target of effective care of HIV infection. Transition to adult care centers provides a major opportunity to optimize management, and this should be regarded as a purposeful process in which short- and long-term goals can be constantly reassessed (22).

We designed an integrated multifaceted intervention with the aim of taking advantage of transition to introduce a medical–psychological intervention and tested it in a pilot study. Our data showed that at the end of the intervention, all the assessed outcomes, and immuno-virological psychological parameters improved; and also, knowledge of the disease improved. The number of patients with CD4+ count above 25% and the number of viro-suppressed patients increased, probably as a result of the simplification of therapy upon application of adult patient schemes. We do not know which intervention among those applied was more effective. The sample size was limited, and the individual conditions were rather heterogeneous. The effects were also affected by family settings. However, the improvement was evident, and many, although not all, unresolved problems improved in all patients.

The interviews provided a worrying scenario with most adolescents not being aware of the peculiar features of the infection and its transmission. Many patients were not aware of sex transmission and protection opportunities. This finding suggests that their attention is mainly focused on themselves and not on their partners and that HIV-infected adolescents need more information about the disease.

However, after intervention, the number of patients with severe distress decreased significantly as indicated by an increase of their global wellness index score. It is probable that the transition process and the cooperation between PID and AID specialists positively affected the sense of safety of patients, lowering the degree of anxiety related to the change of the health-care team and place of care. Although at baseline 38% of patients showed a “severe” level of distress, the majority of adolescents showed a fair degree of self-esteem, having reached or exceeded a score higher than 85, and no further increase was observed at the end of the intervention. Recent data show that relational self-esteem is positively associated with psychological well-being and indicate a connection between relational self-esteem and social support among vulnerable children such as HIV-infected children and adolescents (23). The high self-esteem observed in our population before the intervention may derive at least in part from the psychological support, which is routinely provided by the pediatric center team. A progressive ability of self-management associated with increase in awareness makes the transition to an adult care center easier.

The positive effect of the transitioning program on both viro-immunological and psychological outcomes was maintained for at least 6 months after transition, when the patients were followed up by AID. All adolescents maintained good clinical and immuno-virological control of the disease, and there was no case “lost to follow up.”

The major limitations of the study are the small sample size and the absence of comparative group. Despite these limitations, the results of this pilot study demonstrate for the first time the efficacy of a multifaceted transition process in HIV-infected adolescents and provide a conceptual framework for randomized controlled multicenter trials in larger populations.

In conclusion, transition to the adult health-care system is a delicate step that may lead to a clinical improvement of the disease and also decreasing psychological distress, optimizing resilience, which is a major resource in HIV infection. An integrated multifaceted intervention jointly conducted by pediatricians and adult physician and by expert psychologists is effective in increasing the knowledge of HIV infection, controlling the degree of distress experienced, and providing information about sexual precautions. Finally, it improves the quality of life of vertically acquired HIV adolescents, providing relief to the psychological problems that are often neglected by a “pure” medical care.

## Data Availability Statement

The datasets generated for this study are available on request to the corresponding author.

## Ethics Statement

The studies involving human participants were reviewed and approved by Ethical Committee of the University Federico II of Naples. Written informed consent to participate in this study was provided by the participants' legal guardian/next of kin.

## Author Contributions

EB, GC, AL, and AG made substantial contributions to the conception or design of the work or the acquisition, statistical analysis, or interpretation of data for the work. AG drafted the work or revised it critically for important intellectual content. CR, CS, CM, and FB organized the database and acquired and analyzed the data for the work. GP and MC made contributions to the conception or design. All authors contributed to manuscript revision, read, and approved the submitted version.

## Conflict of Interest

The authors declare that the research was conducted in the absence of any commercial or financial relationships that could be construed as a potential conflict of interest.
